# Deterministic placement of ultra-bright near-infrared color centers in arrays of silicon carbide micropillars

**DOI:** 10.3762/bjnano.10.229

**Published:** 2019-12-05

**Authors:** Stefania Castelletto, Abdul Salam Al Atem, Faraz Ahmed Inam, Hans Jürgen von Bardeleben, Sophie Hameau, Ahmed Fahad Almutairi, Gérard Guillot, Shin-ichiro Sato, Alberto Boretti, Jean Marie Bluet

**Affiliations:** 1 School of Engineering, RMIT University, Melbourne, Victoria 3001, Australia; 2 Univ Lyon, INSA Lyon, CNRS, INL, UMR5270, F-69621 Villeurbanne, France; 3 Dept. of Physics, Aligarh Muslim University, Aligarh, U.P. 202002, India; 4 Sorbonne Université, Campus Pierre et Marie Curie, Institut des Nanosciences de Paris, 4, place Jussieu, 75005 Paris, France; 5 National Institutes for Quantum and Radiological Science and Technology, Takasaki, Gunma, 370-1292, Japan; 6 Department of Mechanical Engineering, College of Engineering, Prince Mohammad Bin Fahd University, Al Khobar, 34754, Saudi Arabia; 7 Faculty of Applied Sciences, Ton Duc Thang University, Ho Chi Minh City, 758307, Vietnam

**Keywords:** color centers, micropillars, proton irradiation, quantum sensing, silicon carbide, vacancy

## Abstract

We report the enhancement of the optical emission between 850 and 1400 nm of an ensemble of silicon mono-vacancies (V_Si_), silicon and carbon divacancies (V_C_V_Si_), and nitrogen vacancies (N_C_V_Si_) in an n-type 4H-SiC array of micropillars. The micropillars have a length of ca. 4.5 μm and a diameter of ca. 740 nm, and were implanted with H^+^ ions to produce an ensemble of color centers at a depth of approximately 2 μm. The samples were in part annealed at different temperatures (750 and 900 °C) to selectively produce distinct color centers. For all these color centers we saw an enhancement of the photostable fluorescence emission of at least a factor of 6 using micro-photoluminescence systems. Using custom confocal microscopy setups, we characterized the emission of V_Si_ measuring an enhancement by up to a factor of 20, and of N_C_V_Si_ with an enhancement up to a factor of 7. The experimental results are supported by finite element method simulations. Our study provides the pathway for device design and fabrication with an integrated ultra-bright ensemble of V_Si_ and N_C_V_Si_ for in vivo imaging and sensing in the infrared.

## Introduction

Silicon carbide (SiC) is an established material for many electronic devices and has also been considered for photonics applications recently. After the improvement of the purity of the material and the isolation of point defects (primarily vacancies), SiC has been considered to host physical systems for quantum devices such as single-photon sources and spin–photon interfaces for quantum interconnects [1–3]. Points defects or color centers in SiC are considered as alternative candidates for quantum applications such as solid-state quantum bits [4–5], spin–photon interfaces [6], single-photon sources (SPSs) [7–10], nanoscale magnetic or electric fields sensors, and pressure or temperature sensors [3,11–13]. Electrically driven SPSs in SiC have been realized [14–16], and the coherent control of electron spin can be achieved up to 500 K [17]. SiC offers an alternative platform for the integration of these quantum systems in large-scale complementary metal-oxide semiconductor (CMOS)-compatible wafers. Also, SiC is suitable for nanofabrication [18–19], and can be controlled through its electronic and piezoelectric properties. Further, it has great potentials for integrated quantum photonics as it has been identified as next-generation photonics material, owing to its second-order nonlinearity, low two-photon absorption, and wide optical transparency. However, there are still many challenges regarding both material fabrication and generation of color centers for integrated photonics applications.

Most of SiC point defects were found more than one decade ago with methods based on measuring ensemble photoluminescence (PL) [20], i.e., the light emission after the absorption of photons, and electron paramagnetic resonance (EPR) [21], which reveals unpaired electrons. Current studies aim to determine more accurately the quantum properties of these defects by using more recent methods of single-photon detection and single-color center isolation, and quantum coherent spin control. The parameters used to describe the quantum properties of color centers include zero-phonon line (ZPL) [22], Debye–Waller factor (DWF) [23–24], zero-field-splitting (ZFS) [25] and optically detected magnetic resonance (ODMR) [26]. The ZPL and the phonon sideband together determine the line shape of individual light-absorbing and emitting molecules [22]. The DWF describes the thermal motion attenuation of X-ray or coherent neutron scattering [23–24]. The DWF can be estimated as the ratio of the ZPL PL emission, compared to the total PL emission, which is the combination of the ZPL PL emission and the phonon-broadened PL. The ZFS refers to the lifting of degeneracy in the absence of a magnetic field. Unpaired electrons interact to give two or more energy states [25]. ODMR is a technique to optically pump the electron spin state of a crystal defect for spin initialization and readout [26].

There are over 200 polymorphs of SiC, and the most relevant are cubic SiC (3C) and hexagonal (4H and 6H), which have different materials properties. The most extensively studied point defects for the quantum technologies described above, specifically for the spin–photon interface, appear in hexagonal 4H-SiC, namely the silicon monovacancy and the silicon and carbon divacancy (V_Si_, V_Si_V_C_) [27]. The V_Si_^(−)^ [28] has two ZPLs, V1/V1′ at 862 and 859 nm, and V2 at 917.5 nm in the 4H polymorph. It has been established to have a spin number of 3/2 and a negative charge state [29–30]. These lines correspond to the two inequivalent cubic (V2) and hexagonal (V1/V1′, with V1′ as a second excited state) sites in 4H-SiC. The intensity of the largest PL from a single defect at saturation is 10 kcts/s [8] without a solid immersion lens using an a-Si detector with 20–30% quantum efficiency and 40 kcts/s with a solid immersion lens [4]. The DWF is 40%, the ZFS is 70 MHz for V2 and 4 MHz for V1 at the ground state [31]. The maximum optically detected magnetic resonance (ODMR) signal (|ΔPL/PL|) after off-resonant optical excitation is 0.04. After resonant optical excitation it is close to 100% [32]. By using the V1 line of the V_Si_, it has been demonstrated that its optical resonances are stable with near-Fourier-transform-limited linewidths, allowing for the exploitation of the spin selectivity of the optical transitions for spin–photon entanglement [33]. In addition to millisecond-long spin coherence times originating from the high-purity crystal, high-fidelity optical initialization and coherent spin control permitted to achieve coherent coupling to single nuclear spins with ca. 1 kHz resolution [27].

The divacancy in 4H-SiC is a neutral charge state defect with spin *S* = 1 with 4 ZPLs PL1–PL4, in the range of 1078–1132 nm [34]. The largest PL intensity is 27 kcps [5] at low temperature using a superconducting nanowire detector with 80% quantum efficiency, and the DWF is 5%. The ZFS is 1.2–1.4 GHz in the ground state and 0.75–0.95 GHz in excited states. Both V_Si_ and V_Si_V_C_ have been proved to be useful for a high-fidelity spin–photon interface [6,27]. PL spectroscopy and EPR measurements combined with ab initio simulations of the nitrogen vacancy, N_C_V_Si_^(−)^ (NV), defect in SiC have been recently performed in [35–38], resulting in ZPLs at 1242, 1241, 1223 and 1180 nm [38]. This emission is very promising as it is further into the infrared compared to the divacancies. The association of ZPL lines with NV centers was eventually demonstrated in [39]. The EPR spin number is 1 and the ZFS is 1.3 GHz. The NV in SiC has also been deliberately created by using ion irradiation in n-type and intrinsic 4H-SiC, showing it can be successfully fabricated [40].

As most of the emitters with spin properties in SiC are in the near-infrared, there is a need to improve the spontaneous emission rate for room-temperature applications such as magnetic sensing and SPSs. For all the above applications, the fluorescence emission enhancement of the color centers is a crucial issue for room-temperature applications, specifically for the V_Si_ and the nitrogen vacancy (N_C_V_Si_) [36].

Micropillars in other materials have been successfully used as photonics structures designed to improve the photoluminescence performance of quantum dots for strong coupling of the emission with the photonics cavity, high photon-extraction efficiency and SPS on-demand generation with near-unity indistinguishability [41–43]. Silicon carbide micropillars have been used in unrelated mechanical property studies in the past [44–45]. Towards the abovementioned goals, a scalable approach for the design and fabrication of photonics wafers to control the local density of states of the emitters is needed. So far, other photonics structures in SiC have been shown, such as photonic crystal cavities in cubic SiC for enhancing the emission of the divacancy V_Si_V_C_ and of V_Si_ [18,46–47], microdisks for enhancing visible interface defects [48–49], nanopillars in 4H-SiC formed by reactive ion etching (RIE) for the improvement of the V_Si_ emission collection efficiency [50], and the use of a solid immersion lens (SIL) for an enhancement factor of three of single V_Si_ [4]. Recent results of the successful enhancement of V_Si_ in 4H-SiC based on nanopillars [50] provided an equivalent enhancement (factor of three) of SIL but with a smaller footprint and better scalability.

The sensitivity of quantum magnetic sensing using spin carrying color centers undergoing ODMR as probes, such as V_Si_, is currently of the order of δ*B* ≈ 10μT/√Hz and can be improved in isotopically pure SiC to δ*B* ≈ 10nT/√Hz [51–52]. By increasing the photoemission collection efficiency (*C*) of the color centers and the number of emitters (*N*), as for example in micropillars, the magnetic field sensitivity can be dramatically improved to reach magnetic sensing resolution of δ*B* ≈ 10nT/√(*C*·*N*·Hz) [53].

Here, we focus on the enhancement of the emission of the V_Si_, V_Si_V_C_ and N_C_V_Si_/4H-SiC platforms aiming at increasing the photon collection efficiency of many emitters to improve the resolution of quantum sensing in biomedical imaging applications due to the favorable emission in the near-infrared. We show the fabrication of micropillars with integrated V_Si_, V_Si_V_C_ and N_C_V_Si_ defects to enhance spontaneous emission and collection efficiency. Compared to earlier works on applications of these materials in quantum technologies, here we focus on the formation of an ensemble of color centers at a defined position in the micropillars, rather than at a random location, for the most extensively studied emitters in SiC useful for spin–photon interfaces. The micropillars, although not yet optimized for an optimal collection of specific color centres, are yielding an ultra-bright emission beneficial for quantum sensing [54] applications using SiC.

## Experimental

### Micropillar fabrication and color center generation

**Figure 1:**
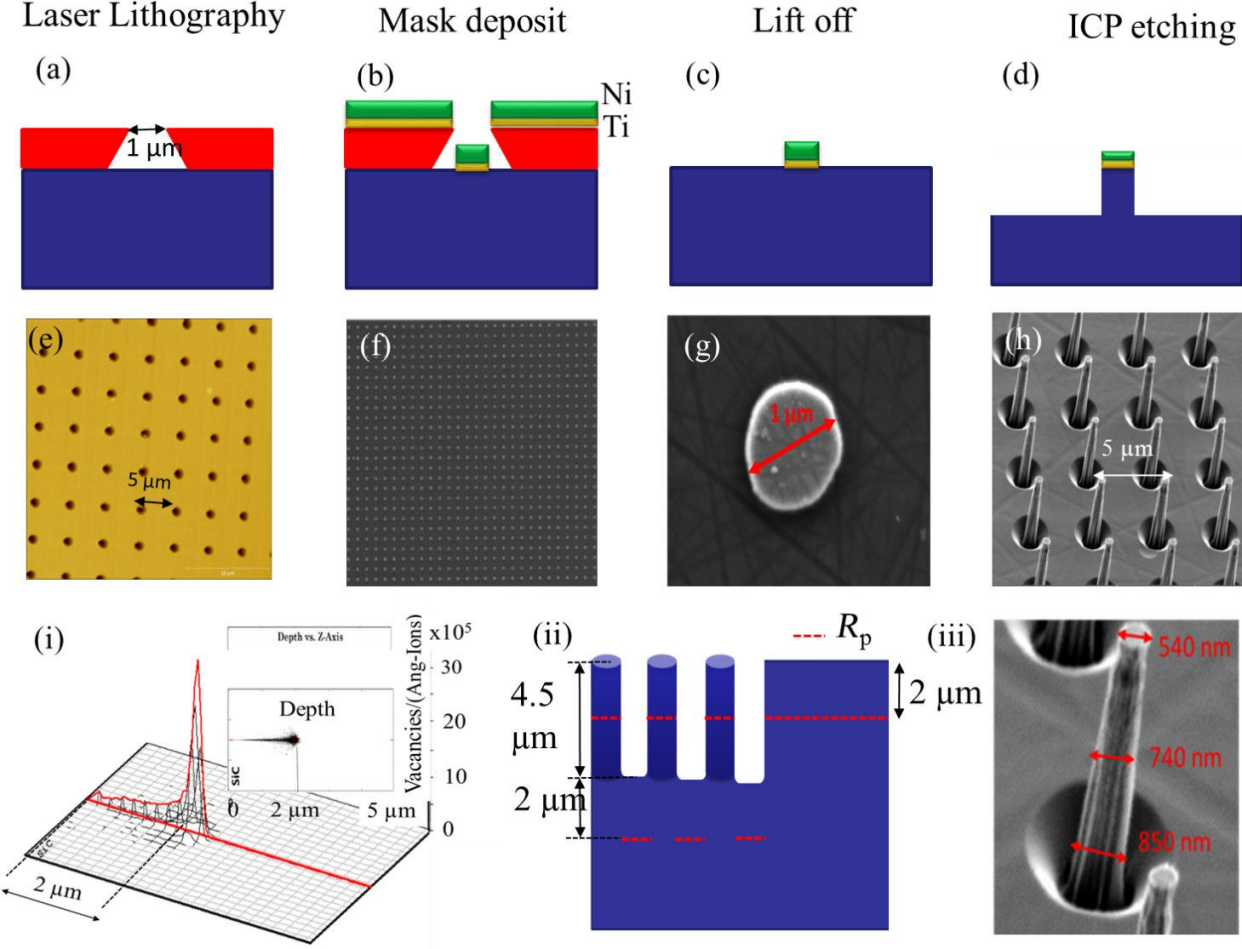
(a–d) Fabrication steps to obtain the 4H-SiC micropillars. (e–h) Optical microscopy and SEM images of various steps highlighted in the top row. (i) Proton irradiation SRIM simulation, with H^+^ stopping line (*R*_p_); (ii) schematics of the vacancy locations in the material. (iii) SEM image of a single pillar.

In this work, micropillars were fabricated by inductively coupled plasma RIE (ICP-RIE) on commercial n-type 4H-SiC. Highly doped n-type 4H-SiC acquired from SiCrystal was used. Using UV laser lithography and a metallic hard mask (Ti/Ni), we have realized micropillar arrays with a height of 4.5 µm, a diameter of 700 nm, and a pitch of 4 µm. The fabrication process (Figure 1a–d) started with coating the samples with photoresist (AZ5214E) and exposing them to a UV laser (λ = 365 nm, Heidelberg µPG101). After that 5 nm titanium and 100 nm nickel were deposited as a metallic hard mask. After that, the samples were etched by ICP-RIE to produced arrays of micropillars.

The samples were then irradiated at room temperature with 300 keV H^+^ at different fluences (10^16^ cm^−2^ and 10^13^ cm^−2^) to create point defects. The maximum H^+^ concentration was simulated to be at 2 µm under the sample surface. Annealing at different temperatures under neutral N_2_ atmosphere was used to promote the presence of different defects (V_Si_ without annealing, 750 °C for 30 min for V_Si_V_C_, 900 °C for 30 min for N_C_V_Si_). The following samples were fabricated and characterized: Samples 1,2 and 3 (H^+^ fluence: 10^16^ cm^−2^) were not annealed and annealed at 750 and 900 °C, respectively. Sample 4 (H^+^ fluence: 10^13^ cm^−2^) was annealed at 700 °C. A summary of the sample treatments is provided in Table 1.

**Table 1:** Description and predominant PL-measured color centers of each sample.

	sample 1 (S1)	sample 2 (S2)	sample 3 (S3)	sample 4 (S4)

fluence of 300 keV irradiation (H^+^ ions/cm^2^)	10^16^	10^16^	10^16^	10^13^
annealing temperature (°C)	—	750	900	700
color centers formation	V_Si_ (V1, V2)	V_Si_, V_Si_V_C_	V_Si_V_C_, N_C_V_Si_	V_Si_ (V1, V2)

### Optical spectroscopy

Room-temperature micro-PL was performed using the Micro-Raman spectrometer LabRAM HR Evolution from HORIBA, equipped with a 785 nm laser. The laser is focused via a 100× 0.9 NA objective on the pillars and on the area with no pillars (gap) at the irradiation depth. The emitted photoluminescence is collected with the same objective and spectrally resolved in a spectrometer in the Czerny–Turner configuration. A Peltier-cooled silicon-based charge-coupled device (CCD) is used as a line-detector in the wavelength range from 200 to 1050 nm. A symphony II InGaAs detector cooled with liquid nitrogen to −110 °C is used for the wavelength range from 800 to 1600 nm.

**Figure 2:**
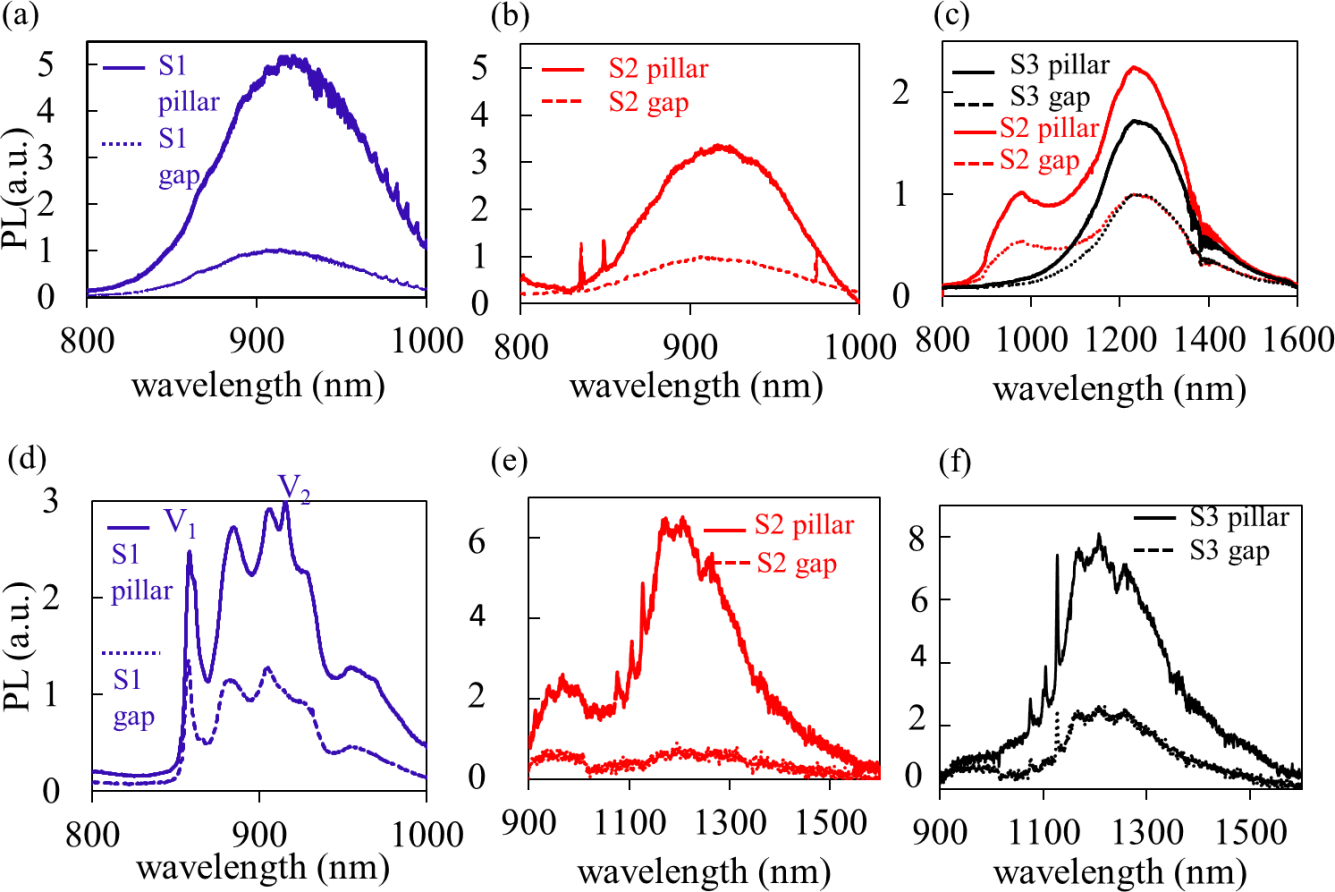
(a–c) Room-temperature PL spectra of the pillars and the gap area (lower dashed curves) for samples S1–S3 excited by a 785 nm CW laser. The PL curves are normalized to the maximum of the gap emission to show the enhancement. The red PL spectra in (c) refer to sample 2 and show the formation of an additional emission other than V_Si_ with a peak at ca. 1300 nm. (d–f) Low-temperature (12 K) PL spectra of the pillars and gaps (lower dashed curve) of samples S1–S3 excited with 100 W/cm^2^ 671 nm CW laser. In (d) the V1 and V2 lines [30] of V_Si_ are visible and the PL curves are normalized to the V2 line in the gap region. In (e) and (f), the PL1, PL2, and PL3 lines [34] of V_Si_V_C_ are clearly seen for the pillars, and the PL curves are not normalized.

The PL emission of the samples S1–S3 after 785 nm excitation (spot diameter 436 nm) at room temperature is shown in Figure 2a–c, respectively; the PL emission after 671 nm excitation (spot diameter 250 nm) at 12 K in shown in Figure 2d–f. The room-temperature PL shows an enhancement by a factor of up to 5.5 for V_Si_ in sample 1 and by a factor of 2.3 for V_Si_V_C_ in sample 3. Our results also confirm the conversion of V_Si_ to V_Si_V_C_ during annealing [55] from sample 2 to sample 3. The enhancement of V_Si_ in sample 2 appears to be lower than that in sample 1.

The low-temperature macroscopic PL spectra show the ZPLs of V_Si_ and V_Si_V_C_ and an important enhancement of the V_Si_ and V_Si_V_C_ PL lines in the region where the pillars are present. It is to be noted that the gap region has also been ion-irradiated, because the irradiation was carried out without mask after the fabrication of the pillars.

**Figure 3:**
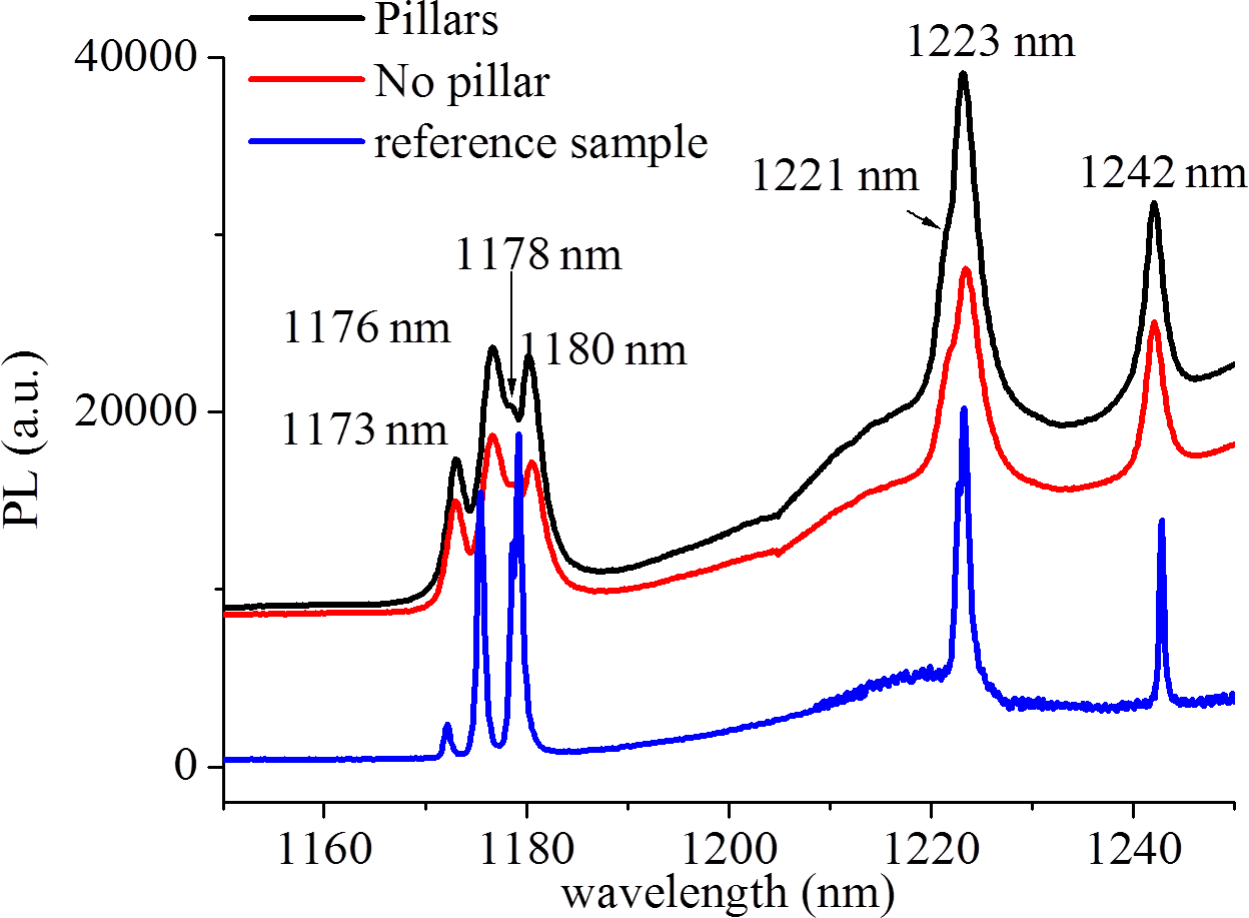
PL emission of sample 3 after excitation at 940 nm at 12 K, showing ZPL emissions at 1221, 1223, and 1242 nm [35,38–39] attributed to NV in 4H-SiC in the micropillars in addition to the ZPLs of the divacancy [34]. The ZPLs from 1173–1180 nm are due to V_C_V_Si_. The spectrum referred to as reference sample was characterized in [38–39] and is given here to prove the emission is indeed due to NV in 4H-SiC.

For excitation at 940 nm the PL spectra of sample 3 shows the activation of the N_C_V_Si_ emission at low temperature [35] after annealing at a higher temperature (Figure 3). Only sample 3 at 940 nm excitation is shown here, as it is most exemplary for the formation of the V_Si_V_C_ and N_C_V_Si_. A detailed confocal microscopy study of N_C_V_Si_ in the pillars is given in the next section. A summary of the PL-measured color centers in each sample is reported in Table 1.

### Confocal microscopy

**Figure 4:**
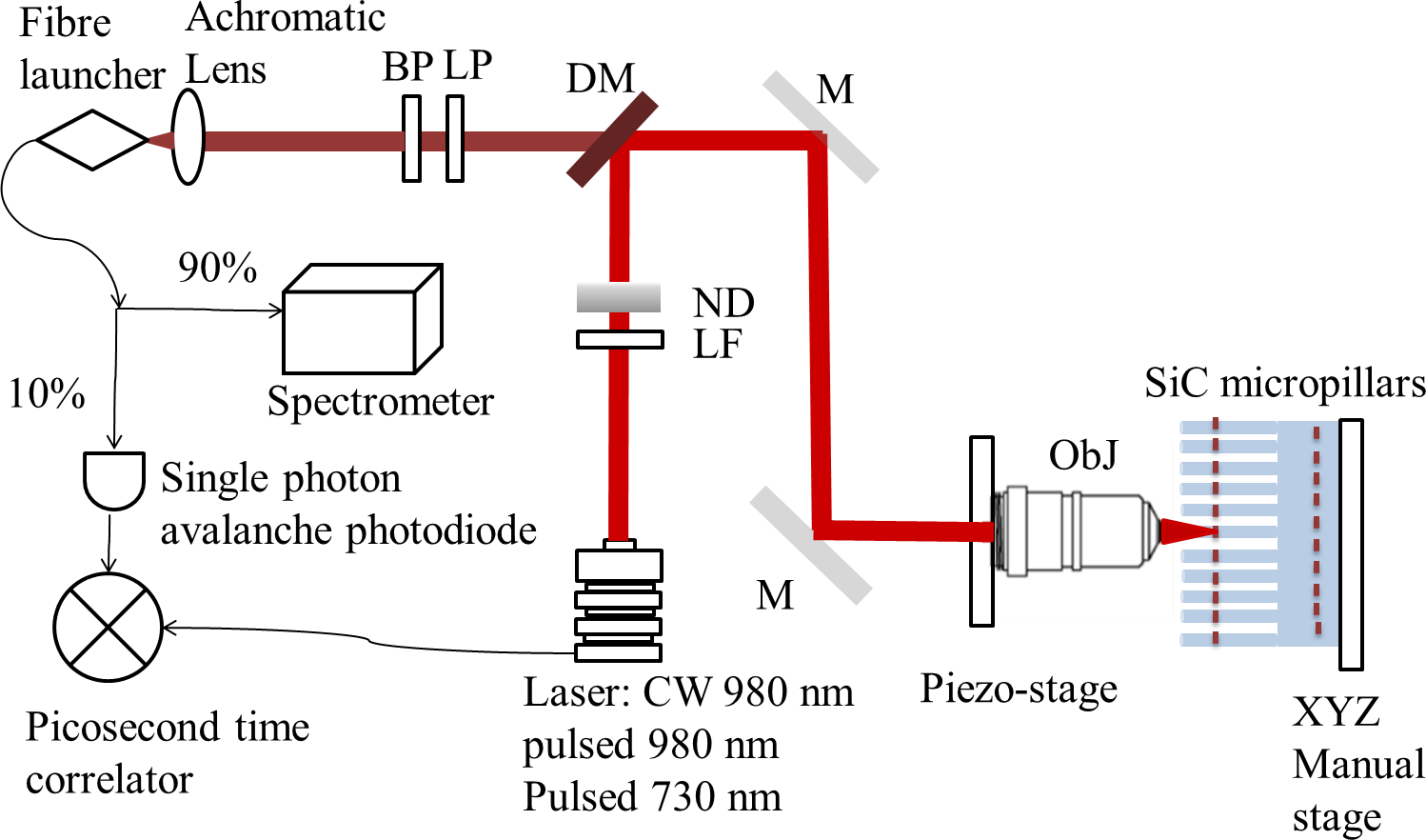
General schematics of the confocal systems used for imaging, spectroscopy and lifetime measurements. The schematics of the SiC micropillars sample is magnified and not to scale for illustration purposes.

Confocal fluorescence scanning microscopy was performed using two custom-built systems, one equipped with Si active quenching avalanche single-photon detectors for measuring the V_Si_ emission and one equipped with InGaAs avalanche single-photon detectors (ID-Quantique 230 Peltier cooled to −90 °C, quantum efficiency maximum of 25% at 1300 nm and set at 10% efficiency during measurement, and dead time set at 2 μs) to measure the N_C_V_Si_ emission (Figure 4).

In the first confocal microscopy setup a variable wavelength super continuum NKT Photonics, Fianium WhiteLase pulsed laser was used for sample illumination at 730 nm. The laser has a pulse width of 30 ps and a repetition rate variable from 10 MHz to 80 MHz. The excitation wavelength to perform the photo counts, photo stability, and power saturation measurements was set to 730 nm (25 nm bandwidth) and 80 MHz. For lifetime measurements we used 40 or 10 MHz. The laser power was finely controlled using a neutral density filter wheel (ND). An Olympus dry objective (100×, 0.85 NA) LCPLN-IR with 85% transmission at 900 nm was used. The objectives were mounted on a PI XYZ computer-controlled stage with an XYZ closed-loop positioner with 200 μm travel in each direction and a step size resolution of 1 nm. The samples were mounted on an XYZ manual stage. The in-plane optical resolution was approximately 500–600 nm at 730 nm excitation. The excitation laser was reflected by a dichroic mirror (DM) single edge at 785 nm (Di02-R785-25x36 from Semrock, transmission >93% between 804.3 and 1200 nm) and the collected photoluminescence signal was transmitted back to the detection arm and filtered using a Thorlabs 850 nm longpass filter (LP, FEL0850). The fluorescence signal was collected using an achromatic aspherical converging lens of 100 mm focal length and sent using a fiber launcher to a multimode 1 m patch fiber coated from 700 to 1500 nm, with 62.5 μm core used as an aperture. The photons are then sent to a single-photon active quenching Si avalanche photodiode. The system is also equipped with a Timeharp260 correlator card from Picoquant to measure the optical lifetime of the emitters.

The second confocal microscopy setup is similar to the one described above with all optics optimized for IR emission. A CW 980 nm diode laser was used to excite the sample and perform confocal mapping, a DM (Semrock Di02-R1064-25x36, filters are LP Semrock BLP02-1319R at 1319 nm and a band-pass filter (BP) from Edmund optics at 1350 ± 50 nm were used to collect the emission signal from N_C_V_Si_. PL is measured in the IR using 90% of the emission from the color centers using a Spectrometer Princeton with a PyLoN-IR camera cooled with liquid nitrogen to −110 °C. A femtosesond 80 MHz pulsed Laser Insight x3 Spectra-Physics at 980 nm was used to measure the lifetime of the NV centers.

## Results and Discussion

### Confocal imaging

**Figure 5:**
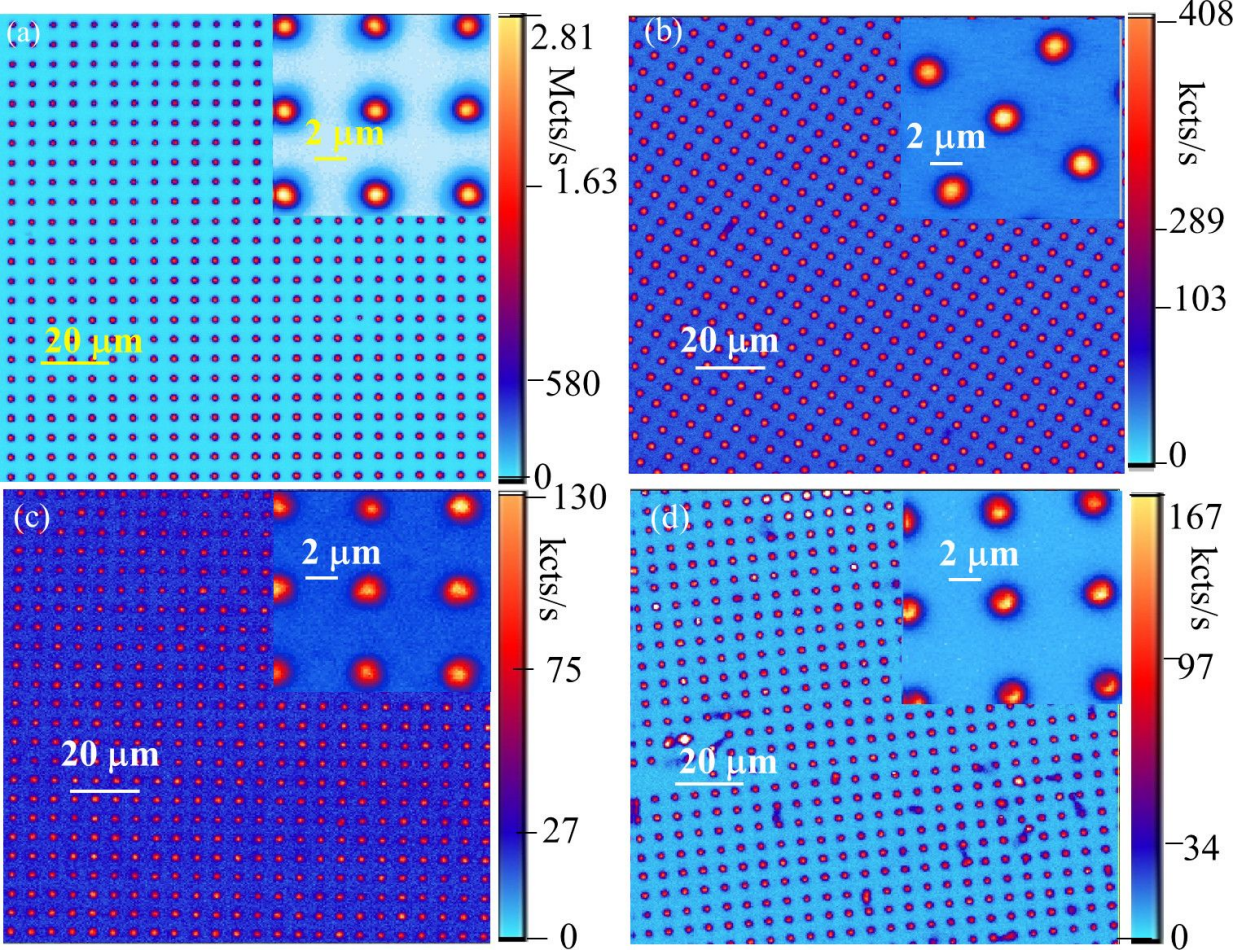
Confocal image and zoom image of a 10 × 10 µm^2^ V_Si_ fluorescent micropillar array excited by a 730 nm laser. (a) Sample 1 excited by 30 µW laser power. (b) Sample 2 excited by 0.7 mW laser power. (c) Sample 3 excited by 1.5 mW laser power. (d) Sample 4 excited by 4.3 mW laser power. An Olympus 0.85 NA infrared objective was used.

Typical confocal images of micropillars in the different samples are shown in Figure 5 when excited at 730 nm, with a focal spot diameter of ca. 429 nm. The confocal microscope can perform up to 200 × 200 μm^2^ image scans, while in Figure 5 we show images of ca. 20 × 20 micropillars corresponding to about 100 × 100 μm. The bright spots correspond to the individual pillars in the arrays. The fluorescence signal was collected through an 850 nm long-pass filter in order to remove any residual red emission from the 730 nm excitation. The observed fluorescence signal varied depending on the annealing temperature, as only the V_Si_ emission signal was detected, with a very bright emission well above the millions of counts per second at excitations below 100 µW from sample 1. We observed an emission signal of the 160 kcts/s in sample 4, corresponding to many emitters. Based on the results from [56–57] the ion fluence values to create single emitters are in the range from 10^10^ to 10^11^ cm^−2^. Thus, we only have an ensemble of V_Si_ defects. A larger background was observed in samples 2 and 3, compared to samples 1 and 4. This is probably because the annealing may induce another emission with ZPLs around 820 nm and a sideband in our detection region [55].

**Figure 6:**
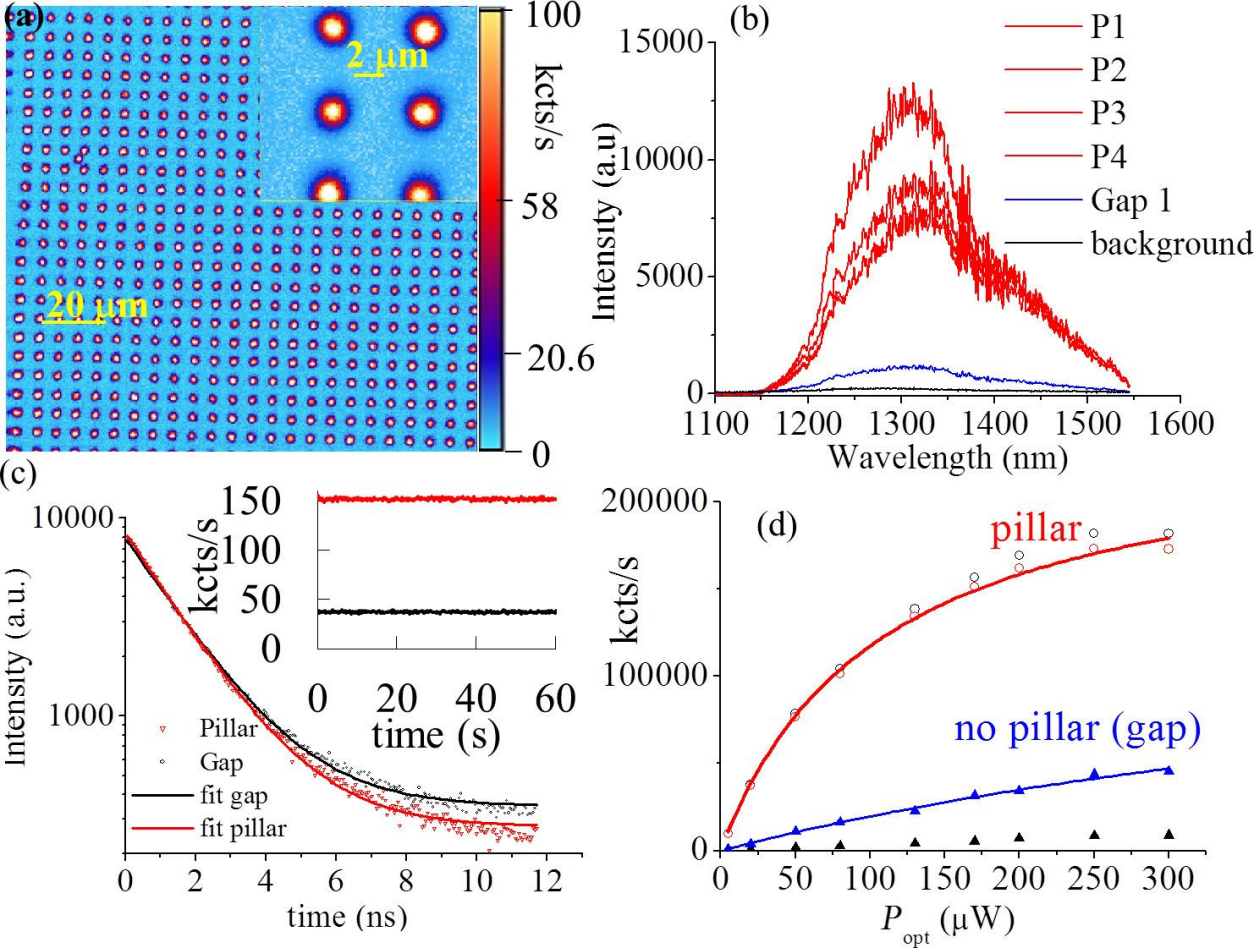
Confocal image and zoom image of a 10 × 10 µm^2^ N_C_V_Si_ fluorescent micropillars array excited by a 980 nm laser, recorded using InGaAs single-photon detectors. An Olympus 0.85 NA infrared objective was used. (a) Sample 3 excited with 100 µW laser power, with only 10% collection emission sent to the single-photon detectors for imaging and integrated count rate, using a LP at 1319 nm and a BP at 1350 ± 50 nm. (b) PL for a few single pillars measured with 90% emission collected by a spectrometer with a InGaAs CCD camera using a LP at 1100 nm. The PL of the pillars is compared with emission from the gap and from the background. (c) Lifetime measurement of a single pillar emission using a LP at 1319 nm and of the emission from the gap. Inset: Photostability of a single pillar emission using a BP at 1350 ± 50 nm and an exaction power of 10.5 μW compared to the gap region. An enhancement by a factor of four is measured. (d) The saturation count rate of one pillar and the gap region, showing the pillar saturation at 108 μW optical power, while the gap region is saturated at 674 μW. The saturation curve is corrected for the background indicated by the black triangles.

A typical confocal image of micropillars in sample 3 (optimized for the formation of the N_C_V_Si_) is shown in Figure 6a during excitation at 980 nm. The bright spots correspond to the individual pillars in the arrays. The fluorescence was collected through a 1319 nm long pass filter and a 1350 nm band-pass filter to primarily collect N_C_V_Si_.

The N_C_V_Si_ emission in the micropillars is very bright. The confocal microscope collects only 10% of the photons, while 90% is sent to the spectrometer (a fiber 10:90 beam splitter was used to image and measure PL at the same time), because each pillar emits around 1 Mcts/s at 100 μW excitation, and the detectors are limited to less than 500000 cts/s due to the long dead time. In Figure 6b the PL spectra of four pillars using confocal microscopy is compared to the emission spectra in the gap and the background arising from the deeper implanted areas.

### Fluorescence photostability, PL enhancement, lifetime

**Figure 7:**
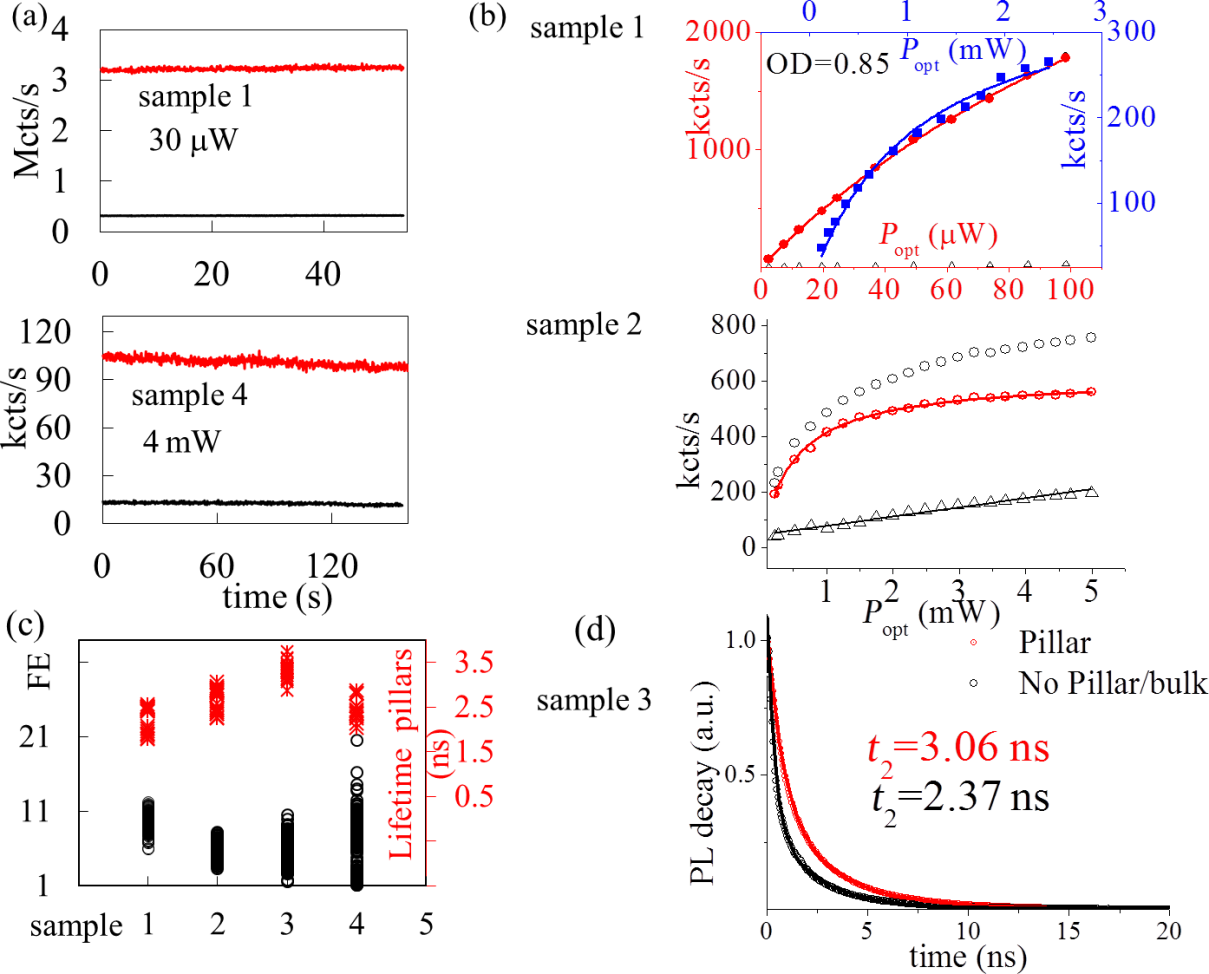
(a) Photostability of one micropillar and the gap region for samples 1 (top) and 4 (bottom) at excitation powers of 30 μW and 4 mW, respectively. Both samples provide a fluorescence enhancement by a factor of about ten. Counts rate are measured every 167 ms. (b) Top: Power saturation curve for one pillar in sample 1 (red) compared with the saturation count rate of the emission in the bulk (blue). The saturation curves show the enhanced emission in the pillar by a factor of 14 and a reduction of the saturation optical power by a factor of 5.6. A neutral density filter with OD = 0.85 was used to perform the measurement. Bottom: Saturation curves for one pillar in sample 2, showing the total count rate, the background, and the signal. (c) Fluorescence enhancement (FE) of more than 100 pillars in different samples (circles) and related lifetime of the emitters in different nanopillars of the four samples. (d) Example of lifetime measurements for V_Si_ in the pillar and in the gap for sample 3, two exponentials decays were observed, the long component of the lifetime is shown. The ratio of the lifetime between the emitters in the bulk and in the pillar (for the longest pillar lifetime measured), τ_bulk_/τ_pillar_, is 0.77.

Photostability measurements showed a photostable emission for all color centers. The inset of Figure 6c shows the photostability of the N_C_V_Si_ centers with an enhancement by a factor of four. However, the emission brightness can be different from pillar to pillar with a maximum emission enhancement by a factor of seven seen in the 1350 nm region. In Figure 7a the photostability of V_Si_ of sample 1 and sample 4 is shown. For each investigated sample with ensemble V_Si_ defects, an excitation laser power-dependent saturation curve of the PL was collected, as shown in Figure 7b for samples 1 and 2. The saturation curve was also studied for N_C_V_Si_ in sample 3. The emission, after background-correcting the count rate, as a function of the optical excitation power *P*_opt_, is modeled by


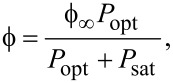


where ϕ is the collected count rate, ϕ_∞_ is the count rate at saturation, and *P*_sat_ is the excitation power at which saturation occurs. For sample 1 the power saturation measurements were performed for the emitters in pillars and for the emitters in the gap, using a neutral density filter of OD = 0.85 (attenuation of a factor of 7.1). In the pillars a saturation count rate of ϕ_∞_ = 5268 ± 185 kcts/s at *P*_sat_ = 194 ± 9 μW was obtained, while in the gap, corresponding to bulk emission, the saturation occurs at the optical power of *P*_sat_ = 1.09 ± 0.09 mW, with a saturation count rate of ϕ_∞_ = 373 ± 14 kcts/s. In sample 1 we observed for one specific pillar an enhancement factor of 14 at saturation in terms of count rate and a reduction of the optical excitation power by a factor of 5.6. For V_Si_ a count rate of 22 kcts/s of a single emitter in a nanopillar with optical pump saturation occurring at 3.5 mW has been shown previously [48]. This corresponds to a fluorescence enhancement (FE) by a factor of two to three compared to single emitters in non-fabricated samples. Thus, for sample 1 we estimate in the gap about ca. 41 emitters, assuming a count rate of a single emitter at saturation of about 9000 cts/s. For sample 2 saturation occurs also at a lower optical power of *P*_sat_ = 485 ± 11 μW with a saturation count rate of ϕ_∞_ = 615 ± 3 kcts/s, compared to the gap with *P*_sat_ = 1.089 ± 0.09 mW with a saturation count rate of ϕ_∞_ = 373 ± 14 kcts/s. In this specific pillar, there is only an enhancement by a factor of 1.7 at saturation. Finally, in a specific pillar of sample 3 *P*_sat_ = 2.5 ± 0.3 mW with a saturation count rate of ϕ_∞_ = 206 ± 15) kcts/s. Samples 2 and 3 were not optimal for V_Si_ enhancement due to the formation of other defects during annealing.

For sample 3 probed using 980 nm excitation and collecting using a 50 nm BP at 1350 nm, a saturation of the IR emission, attributed to N_C_V_Si_, occurs also at a lower optical power of *P*_sat_ = 107.5 ± 0.1 μW with a saturation count rate of ϕ_∞_ = 243 ± 1 kcts/s, compared to the gap with *P*_sat_ = 673.89±0.09 μW with a saturation count rate of ϕ_∞_ = 153 ± 1 kcts/s (Figure 6.d). It is to be noted that the single-photon detectors in the IR are saturating at less than 500 kcts/s due to exceptionally long deadtime (2 μs) as such only 10% of the emitted photons were collected to have enough dynamic to capture saturation.

In Figure 7c the fluorescence enhancement of the V_Si_ emission was measured for many pillars (more than 100) for each sample in the linear low-power pumping regime. Higher enhancements for sample 1 and sample 4 are observed, with an increased enhancement for the sample with the lower irradiation dose.

In Figure 6c the measured lifetimes of N_C_V_Si_ emission using a LP at 1319 nm were τ = 1.557 ± 0.006 ns in the pillar and τ = 1.621 ± 0.006 ns in the gap. The values were modeled using a single exponential. These short lifetimes indicate that the N_C_V_Si_ center can be ideally used as a bright SPS at room temperature.

The lifetime associated with V_Si_ in the gap area was measured as τ = 2.35 ± 0.01 ns, similar for all samples. These values are lower than the lifetimes of 6 ns in intrinsic 4H-SiC [8,58] and of 5 ns in n-type 4H-SiC [59]. This is attributed to material doping reducing the lifetime. The lifetime is higher for the pillars in samples 2 and 3 than for those in samples 1 and 4. We attribute this difference to the annealing, which reduces the number of defects in the crystal. For V_Si_ the lifetime was modeled by two exponential decay functions.

### Modeling of V_Si_ emission in micropillars

We model the emission of color centers in the pillars using the finite-element method COMSOL Multiphysics radio frequency module. A V_Si_ color center is modeled as an oscillating point dipole located along the central axis of the SiC pillar. For calculations we used the index of refraction of the SiC domain, *n*_SiC_ = 2.59, at the assumed V_Si_ emission wavelength of 900 nm (the center is 917 nm). Considering the SEM images of the fabricated SiC pillars, the length of the SiC pillars was set to 4.3 μm. The top radius of the conical SiC pillars was set to 270 nm and the bottom radius to 425 nm. The V_Si_ dipole is assumed to be a linear excitation dipole along the defect axis, i.e., 8° off the *c*-axis, which the micropillars are aligned to. We studied the spontaneous emission of the V_Si_ dipole at different depths along the full length of the pillar. To have a better understanding of the calculated results, we also studied the spontaneous emission of the V_Si_ dipole in cylindrical SiC pillars of varying radii from 160 to 600 nm. Based on these simulations, we calculated the Purcell enhancement, the collection efficiency (CE) enhancement, and the fluorescence enhancement relative to V_Si_ emission in bulk SiC. The Purcell enhancement is obtained in terms of the total power radiated by the dipole in the SiC pillar to that radiated in bulk SiC, after considering the quantum efficiency of the emitting dipole. To obtain the collection efficiency, we measure the power collected on a circular surface at the domain top the diameter of which is chosen such that it acts as a collection lens with a numerical aperture of 0.9 (corresponding to the aperture of the collection objective of the confocal microscope). CE enhancement is then obtained as the ratio between the radiated power collected at the top surface and the radiated power collected on the same surface for dipole emission in bulk SiC. To have a comparison with the experimentally measured PL enhancement, the calculated FE is defined by the equation [60]:


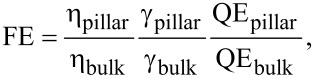


where the color center bulk quantum efficiency, QE_bulk_, and the SiC pillar quantum efficiency, QE_pillar_, are assumed to be the same. Here, η_pillar_/η_bulk_ is the ratio between the collection efficiency in the SiC pillar and that in the bulk (CE enhancement) and γ_pillar_/γ_bulk_ is the ratio between the corresponding excitation/emission rates (Purcell enhancement). The relative excitation rates should be the same as relative emission rates for QE of 1. For QE = 1, the relative emission rate is calculated as the total power radiated by the dipole emitter in the SiC pillar relative to that in bulk SiC [61]. The total emission rate is equivalent to the reciprocal of the total lifetimes,


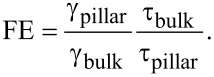


**Figure 8:**
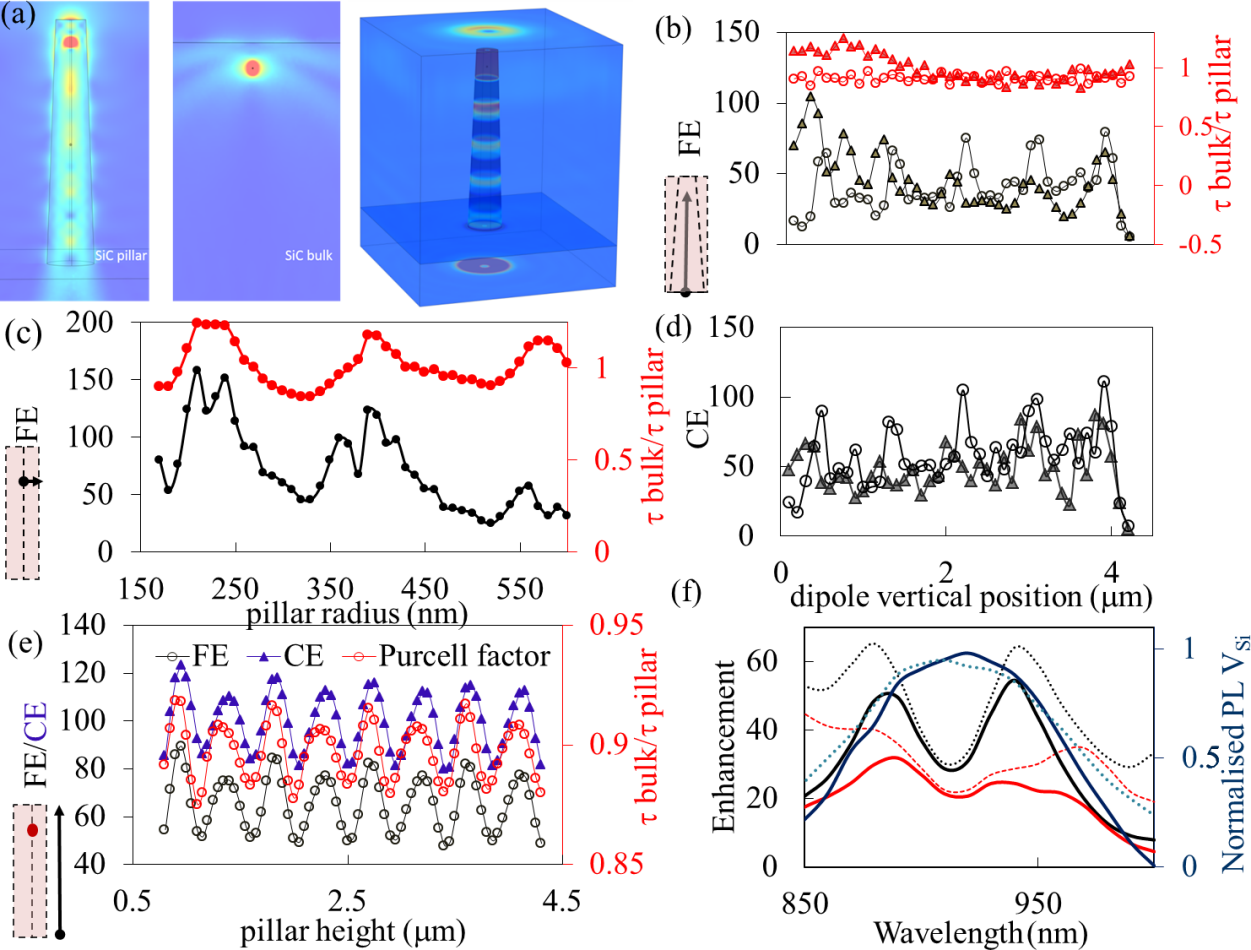
Finite-element method simulation for a pillar of 4.3 µm height and a dipole orientation 8° off the *c*-axis. Two geometries of the pillars are considered, cylindrical (circles) and conical (triangles). (a) Radiation pattern: intensity and power outflow from a conical pillar with emitters at 3.9 μm from the bottom of a pillar, compared to an emitter 400 nm below the bulk surface. (b) FE and τ_bulk_/τ_pillar_ as functions of the vertical position of the dipole for a cylindrical pillar of radius 350 nm (circles) and for the conical pillar (triangles). (c) FE and τ_bulk_/τ_pillar_ as functions of the radius of a cylindrical SiC pillar for a dipole located at 3.9 μm from the bottom. (d) Simulated collection efficiency (CE) enhancement relative to bulk as a function of the vertical position of the dipole in a cylindrical pillar of radius 350 nm (circles) and for the conical pillar (triangles). (e) FE, CE, and Purcell factor as functions of the pillar height of the conical pillar with a fixed emitter at 450 nm from the surface. (f) FE (red lines) and CE (black lines) enhancement as functions of the wavelength for the conical pillar with an emitter located at 2.3 µm from the bottom (experimental case), showing the effect of the broad emission of V_Si_ on FE and CE. The FE reduction across the spectra is ca. 24% compared to a narrow emission. Dashed lines represent the calculated enhancements, and the solid lines represent the enhancement modulated by the emission spectra of V_Si_, which is also shown as a dashed blue line in the bulk and solid blue line in the pillar.

This ratio is almost constant due to the low QE of V_Si_ in SiC, as observed in Figure 8b. The QE of V_Si_ is assumed to be 0.3 from previous measurements of decay associated with intersystem crossing, which were based on the decay rates from the excited state to the metastable state and to the ground state of a single emitter [8]. Other non-radiative decays due to phonon sideband and thermal dephasing are neglected here.

From the collection efficiency calculations, we found the collection efficiency to be substantially enhanced for dipole emission in the SiC pillar. For a dipole oriented 8° off the *c*-axis, the η_bulk_ value is found to be very low, ca. 0.0038. However, in a SiC pillar of 350 nm radius η_pillar_ varies in the range from 0.1 to 0.3 along the length of the pillar. This is more than an order of magnitude enhancement of the collection efficiency, with a possible maximum collection efficiency of 30%.

In the FE calculations, we saw successive periodic peaks in the FE enhancement factor along the length of the pillar, with a spacing between the peaks of 400–450 nm, which is about half of the dipole emission wavelength (Figure 8b, circles). The first peak appears at ca. 400 nm from the top surface (ca. 3.9 μm along the pillar height). The successive nodes of the radiation pattern of the dipole emission can be seen in Figure 8a. When varying the SiC pillar radius, we obtained the best performance for a radius of ca. 210 nm (Figure 8c). This corresponds to a pillar diameter of ca. 420 nm, about half of the dipole emission wavelength. For the optimal radius the FE enhancement factor is ca. 160, corresponding to τ_bulk_/τ_pillar_ = 1.24. A small variation of these results is observed for the conical shape (Figure 8b). The cylindrical model yields information about the optimal radius for fabrication. We also varied the pillar height and observed successive modes separated by ca. 450 nm, about half of the dipole emission wavelength. The optimum pillar height is 0.9 μm (Figure 8e).

Under our experimental conditions (conical shape and emitters at ca. 2.3 μm from the bottom), we predict FE = 29 at 900 nm, which is reduced to 20 because of the broad emission of the center and average values of CE = 31 and τ_bulk_/τ_pillar_ = 0.92. The experimental values vary from pillar to pillar with FE = 3–20 and τ_bulk_/τ_pillar_ = 0.77–0.92. The discrepancy between the experimental results and the simulation results is attributed to the broad emission of V_Si_ of about 100 nm, which significantly reduces the average enhancement as shown in Figure 8f. Additionally, samples 2 and 3 are not optimal for enhancement because of the annealing and the formation of other color centers that can quench the emission. Further, the location of the emitter and the size of the pillar (radius and height) also influence fluorescence enhancement and lifetime. Our results, therefore, show overall a good match between the calculated and experimental values. They are slightly different from [50], where the best position for the emitters, to increase guiding of the nanopillars, is close to the bottom. Here, the SiC pillar height was about 800 nm. Also, peaks in the collection efficiency can be observed at about 400 nm and more from the top pillar surface.

## Conclusion

We have fabricated micropillars with a deterministic placement of near-infrared color centers in SiC using hydrogen irradiation and annealing at different temperatures. We have optimized the enhancement of color centers relevant for quantum sensing applications, such as silicon monovacancies and divacancies, and nitrogen vacancies. Due to the high fluence during implantation, we did not observe single emitters. We achieved a spontaneous emission enhancement by a factor of up to 20 for the silicon monovacancies achieving several Mcts/s in a single pillar from the ensemble of emitters. Based on our results we foresee further optimization and studies of the micropillar design for increased resolution and sensitivity in magnetometry using these emitters [51–53,62]. An optimized design based on finite-element method analysis is also proposed for the enhancement of the V_Si_ defect emission. The optimized design is a pillar with a radius of ca. 210 nm radius and a height of 0.9 μm in which the emitters are localized ca. 450 nm from the top. This design could improve the V_Si_ and V_Si_V_C_ SPSs collection efficiencies [4–5,8] for spin–photon interfaces and quantum network applications [1]. Finally, we have enhanced by a factor of up to 7 the emission of N_C_V_Si_ in SiC, achieving more than 2 Mcts/s photostable emission at 1350 ± 50 nm in a single pillar at room temperature. Due to the remarkably high brightness of the N_C_V_Si_ in SiC and the measured lifetime in the range of ca. 1.6 ns, we expect this defect to be ideal for single-photon generation. In addition, the ZFS of this defect can be optically detected. For both future experiments, FE in micropillars could be beneficial. The development demonstrated here also paves the way to achieve more sensitive quantum magnetic sensing devices, in particular for deep tissue imaging in biological analyses due to the favorable emission of N_C_V_Si_. Deep imaging of biological tissues is a significant challenge as light is scattered by the tissues, distorting the focus of optical microscopes. Imaging at optical super-resolution may be achieved by using novel devices based on the engineered defects in the SiC micropillars. Further optimization of the photonic structure, from quality and quantity of color centers, over unit-specific surface area as well as unit volume, to the size, shape and relative positioning of the micropillars, will be needed for specific applications.
